# Direct recognition of LPS drive TLR4 expressing CD8^+^ T cell activation in patients with rheumatoid arthritis

**DOI:** 10.1038/s41598-017-01033-7

**Published:** 2017-04-19

**Authors:** Archana Tripathy, Shweta Khanna, Prasanta Padhan, Shuchi Smita, Sunil Raghav, Bhawna Gupta

**Affiliations:** 1 School of Biotechnology, Kalinga Institute of Industrial Technology, Bhubaneswar, Odisha India; 2 Department of Rheumatology, Kalinga Institute of Medical Sciences, Bhubaneswar, Odisha India; 3 Institute of Life Sciences, Bhubaneswar, Odisha India

## Abstract

Aberrant immune responses characterize autoimmune disorders like Rheumatoid Arthritis (RA) wherein lymphocytes are recognized as key players. Role of CD8^+^ T cells in RA has been less defined however we found that these cells are activated in RA patients with increased expression of cytolytic granules and inflammatory mediators thereby modulating immune responses contributing to disease severity. Though unconventional expression of different Toll Like Receptors (TLRs) on CD8^+^ T cells has been proposed but their expression and role in T cell activation and differentiation in RA still remains obscure. Herein we report, for the first time, an increased expression of TLR4 on peripheral CD8^+^ T cells of RA patients and its role in skewing CD8^+^ T cells towards activated and inflammatory phenotype thereby playing a significant role in pathogenesis and progression of RA. We found that the surface expression of TLR4 on CD8^+^ T cells directly correlates with disease severity. Moreover, these CD8^+^ T cells respond to the TLR4 ligand LPS and express robust amounts of cytotolytic and inflammatory molecules including TNFα and IFNγ. Our study hence identifies an important role for CD8^+^ T cells in orchestrating RA through TLR4 mediated activation and differentiation.

## Introduction

Rheumatoid arthritis (RA) is an autoimmune disease characterized by abnormal immune responses to self-antigens. Though the pathogenesis of RA is not yet fully elucidated, we and others have shown that it can be induced by environmental factors^1^ on a genetically susceptible background^2–4^. The condition leads to abnormality in antigen recognition and or presentation^5–7^, lymphocyte activation as well as differentiation^8, 9^, thereby resulting in enhanced production of pro-inflammatory cytokines and auto-antibodies eventually causing damage to joints. Though multiple, non-redundant checkpoints are in place to prevent such potentially deleterious autoimmune responses the strong genetic associations between RA and genes encoded within the HLA locus as well as the presence of tissue invading lymphocytes in the lymphoid microstructures^10^, suggests a central role of T cells in the pathogenesis of RA. Role of CD4^+^ T cells in progression of rheumatoid arthritis has been frequently proposed as the key mechanism^11, 12^. Selective expansions of CD4^+^ T cells clones in RA have been repeatedly reported^13, 14^. Differentiation of CD4^+^ T cells into IFNγ producing Th1 and IL17 producing Th17 cells^15^ with a significant loss of Tregs have been implicated in the pathogenesis and progression of RA^16, 17^. Most studies evaluating the balance of T effector subsets have examined CD4^+^ T cells exclusively and less attention has been given to understand the role of CD8^+^ T cells in RA. A few recent studies have begun to address the contribution of CD8^+^ T cells to disease initiation, progression or severity^18^. CD8^+^ T cells are conventionally acknowledged for their significant role in defense against intracellular pathogens. They are shown to rapidly shift from a quiescent to an active state upon re-infection with a pathogen, thereby producing massive amounts of effector molecules like Granzyme and Perforin^19–21^. IFNγ is a central effector molecule of CD8^+^ T cells that has a broad spectrum of activity^22, 23^. IFNγ not only dampens the growth of pathogens, it also recruits neutrophils to the site of infection and activates immune cells such as macrophages to potentiate the innate immune response^24–26^. Kang *et al*. showed that synovial CD8^+^ T cells have certain mechanisms through which they modulate RA outcomes^27^. Recently Carvelheiro *et al*. described different CD8^+^ T cell subsets and their cytokine profile related to RA disease activity^28^. They showed that the CD8^+^ T cells in active RA patients have a cytotoxic behavior and secrete significant amounts of pro-inflammatory cytokines like TNFα. Another study indicates that these cytotoxic CD8^+^ T cells can induce inflammation by activating CD4^+^ effector T cells, with IFNγ producing CD8^+^ T cells inducing Th1 response^29^.

Interestingly, unconventional expression of Toll-like Receptors (TLRs) on T cells has been described^30–32^. TLRs recognize various signature microbial products and are largely known to function in cells of the innate immune system where ligand binding induces maturation of antigen-presenting cells and inflammatory cytokines production^33^. Recent studies have reported the presence and significance of TLRs in CD4^+^ T cells in inflammatory and autoimmune diseases. Studies indicating co-stimulatory role of TLR2 for both CD4^+^ and CD8^+^ T cells^34, 35^, in regulation of IL-17 production by γδ T cells^36^ and CD4^+^ T helper 17 (Th17) cells^37^ are well documented. Expression of TLR3, TLR2 and TLR7 and their function as co-receptors, enhancing TCR-induced IFN-γ secretion upon engagement with specific agonists, has been reported in CD8^+^ T cells^31, 32^. Given the fact that peripheral blood of RA patients banks a number of auto-antigens^38^ and pathogens^39^ that may lead to TLR-induced systemic inflammation by CD8^+^ T cells, we sought to investigate this in more details. To identify differential expression of TLRs on the surface of CD8^+^ T cells in RA patients we first screened the mRNA expression of TLR1 till TLR10 and observed a significant expression of TLR4 on CD8^+^ T cells of RA patients in comparison to the healthy controls (data not shown). We therefore continued to analyze the expression and functional significance of TLR4 on CD8^+^ T cells. We could show that TLR4 is expressed exclusively in naïve, effector as well as effector memory CD8^+^ T cells of RA patients and the expression varies with disease activity. These TLR4 expressing CD8^+^ T cells secrete significant amounts of cytolytic granules like Granzyme B. Moreover, functional assays demonstrate that the engagement of TLR4 by its agonist increases the expression of cytolytic molecules and inflammatory cytokines by CD8^+^ T cells. Taken together, these results clearly indicate CD8^+^ T cells as critical modulators of the disease and LPS engagement of the functional TLR4 expressed on their surface activate these lymphocytes thereby contributing directly to the maintenance of chronic inflammatory processes in RA.

## Results

### Increased expression of TLR4 by CD8^+^ T cells in RA patients

**Figure 1:**
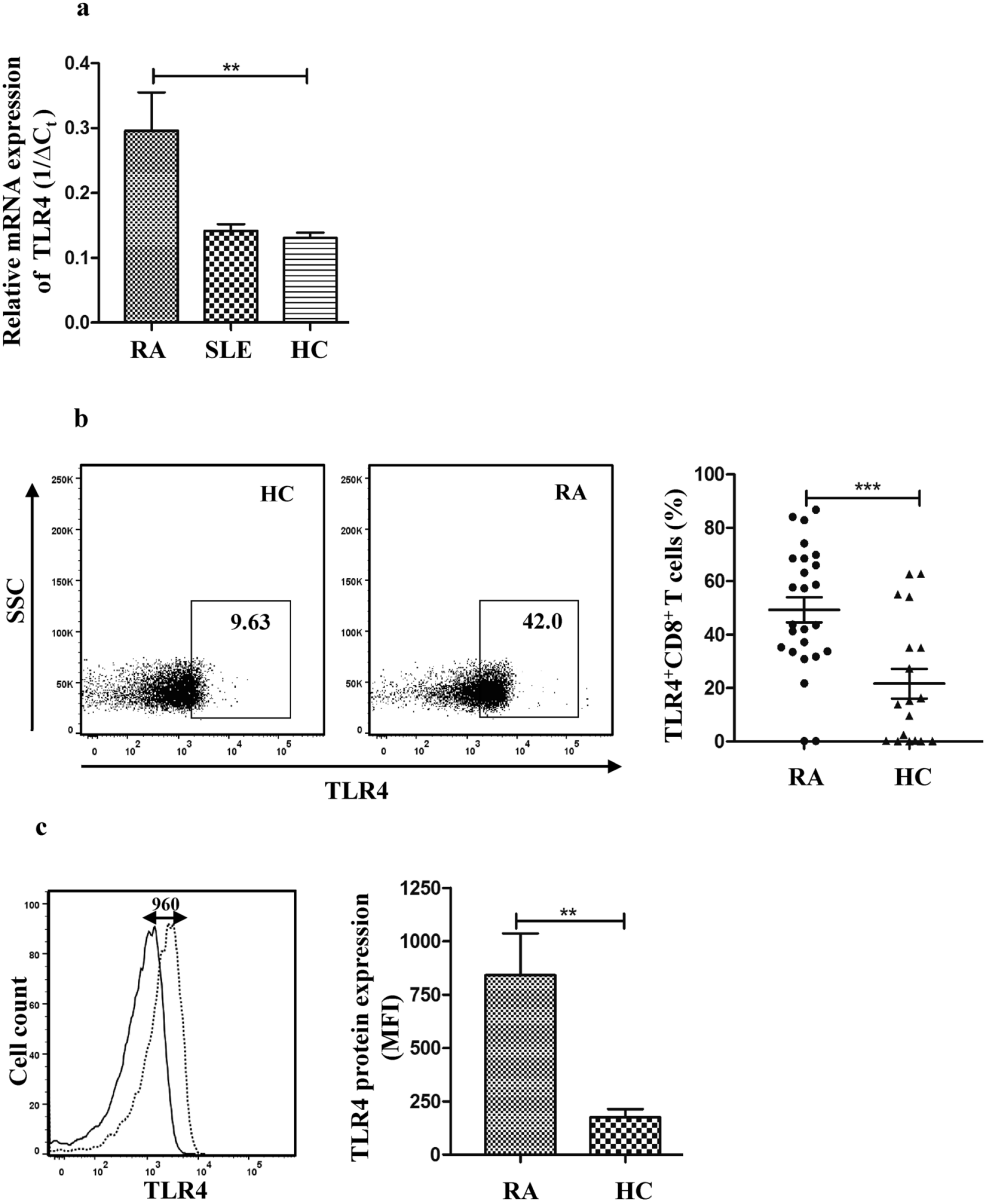
CD8^+^ T cells show increase TLR4 expression in RA patients. (**a**) Relative mRNA expression of TLR4 in peripheral CD8^+^ T cells was determined by qPCR from 45 RA patients, 9 SLE patients, and 42 healthy controls (HC). The qPCR C_t_ values were normalized (∆C_t_) by subtracting the C_t_ values of ß-actin from those of TLR4 and 1/∆C_t_ was plotted. (**b**) A representative FACS dot plot panel shows TLR4 expression by CD8^+^ T cell in a HC and RA patient. The box gate represents CD3^+^CD8^+^ TLR4^+^ T cells. The graph represents percentages of CD3^+^CD8^+^TLR4^+^T cells in our case (n = 25) - control (n = 18) cohort. (**c**) Histogram shows a comparative fluorescence distribution of TLR4 for CD8^+^ T cells from a single HC and RA patient. A shift in the fluorescence intensity represents an increase in TLR4 expression on CD8^+^ T cells of the RA patient. Solid line represents HC and dotted line represents RA. The bar graph represents median fluorescence intensities (MFI) of TLR4 protein expressed on CD8^+^ T cells in the case-control samples as in (**c**), and indicates significant increase in TLR4 expression in RA patients. Bars represent the mean ± SEM. **p < 0.01, ***p < 0.001.

We first set out to check TLR4 expression by CD8^+^ T cells (Fig. 1). The qPCR analysis revealed that CD3^+^CD8^+^ T cells of RA patients expressed significantly increased (*p* < 0.01) amounts of TLR4 mRNA transcripts (18 fold higher) in comparison to the healthy donors (Fig. 1a). We also observed a substantial increase in the TLR4 mRNA transcript expression in RA patients in comparison to the patients with SLE, another autoimmune disorder.

To examine whether TLR4 protein is as well expressed on the surface of CD8^+^ T cells we analyzed TLR4 expression on CD3^+^CD8^+^ T cell population from the whole blood or on isolated CD8^+^ T cells using RosetteSep CD8^+^ T cells enrichment kit from RA patients and healthy donors (HC) using flow cytometry (Supplementary Fig. 1a,b). The percentage of CD8^+^ T cells expressing TLR4 on their surface significantly increased (p < 0.001) in RA patients (49.3% ± 4.7%) in comparison to the healthy controls (21.7% ± 5.5%) (Fig. 1b). A concomitant increase (p < 0.01) in the median fluorescence intensity (MFI) of CD3^+^CD8^+^TLR4^+^ T cells was observed in RA patients (840.8 ± 196.1) in comparison to the healthy donors (174.2 ± 39.8) (Fig. 1c). The differences remained prominent and significant when the TLR4 expression was analyzed either on CD3^+^CD8^+^ T cells directly using whole blood or on CD8^+^ T cells isolated using RosetteSep kit. We also observed TLR4 protein expression on CD3^+^CD8^+^ T cells from synovial fluid of RA patients (17.9% ± 3.63%).

### Surface expression of TLR4 on CD8^+^ T cells increases with disease progression

**Figure 2:**
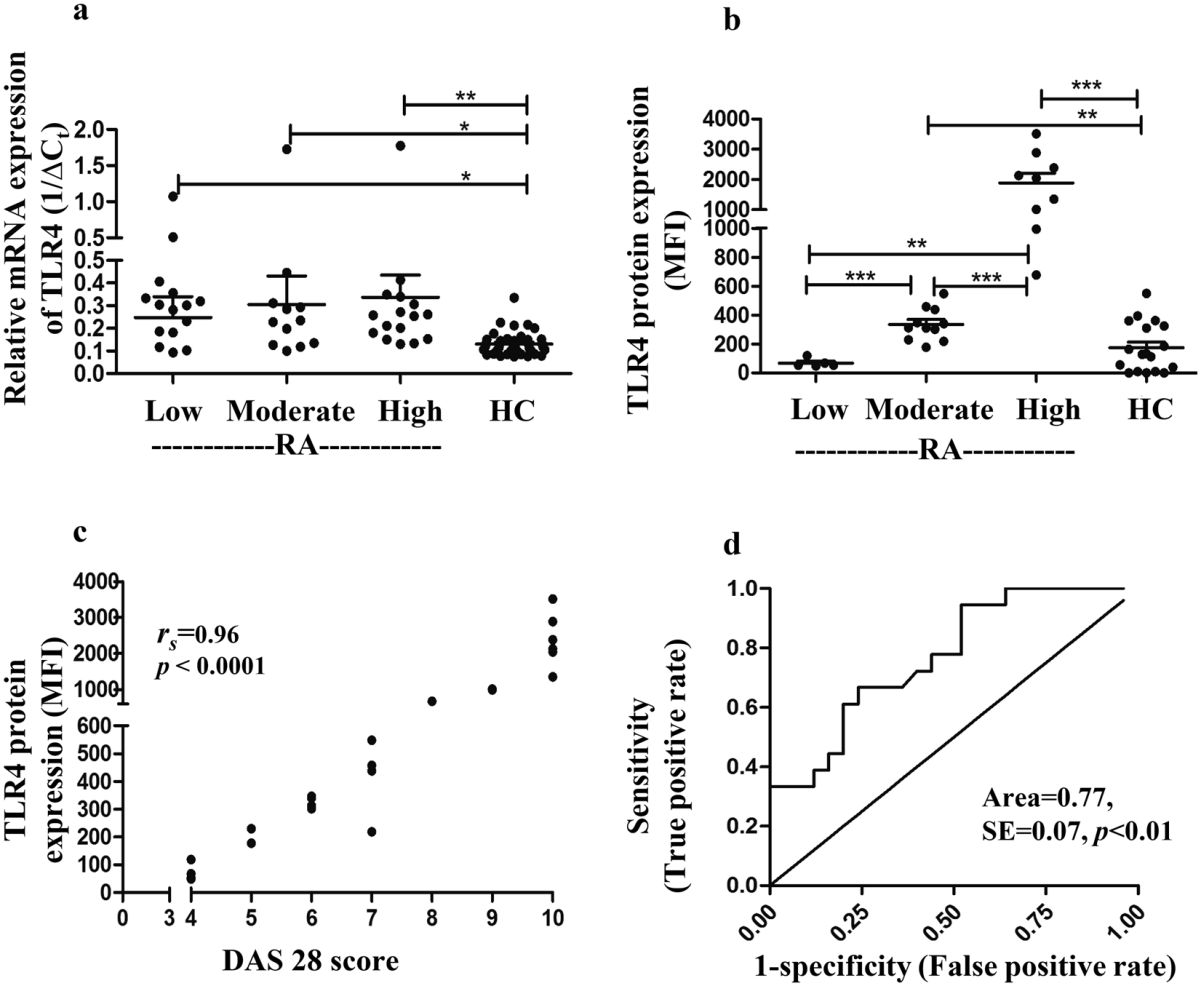
TLR4 expression by CD8^+^ T cells in RA patients at different disease activity states. (**a**,**b**) The scatter plots represent TLR4 mRNA and protein expression by CD8^+^ T cells of healthy controls (HC) and RA patients segregated according to low, moderate and high DAS28 scores. (**c**) The figure shows Spearman non-parametric correlation analysis of TLR4 protein (MFI) with DAS28 score. (**d**) ROC analysis for TLR4 protein expression in RA and HC samples. CD8^+^ T cells from 45 patients (DAS28: low = 16, moderate = 13, high = 16) and 42 controls were analyzed for TLR4 transcript analysis and CD8^+^ T cells from 25 patients (DAS28: low = 5, moderate = 11, high = 9) and 18 controls were analyzed for TLR4 protein analysis. Bars represent the mean ± SEM. *p < 0.05, **p < 0.01, ***p < 0.001, SE = standard error.

DAS28 score for each RA patient was calculated taking into account different clinical symptoms and manifestations to identify disease severity. RA patients were then segregated according DAS28 scores into low (DAS28 ≤ 4), moderate (DAS28: 5–7) and high (DAS28 > 7) disease activity states and their CD8^+^ T cells were analyzed for expression of both TLR4 mRNA transcript and surface protein. Interestingly, a substantial increase of TLR4 mRNA transcript was observed in RA patients even with low and moderate disease activities however, the difference increased statistically (*p* < 0.01) in patients with high disease activity and eventually higher DAS28 scores in comparison to the healthy cohort (Fig. 2a). However we did not observe significant differences in the mRNA transcript expression between the three groups of RA patients.

We next analyzed the cell surface protein expression of TLR4 on CD8^+^ T cells in the three different groups of RA patients (Fig. 2b). TLR4 protein expression on CD8^+^ T cells was significantly higher in all RA patients moreover, unlike mRNA transcript expression, we found significant differences among the three groups of RA patients (Fig. 2a,b). Intriguingly, RA patients with low DAS28 scores did not show significant changes in the TLR4 protein expression on CD8^+^ T cells in comparison to the healthy donors. However, TLR4 protein expression was significantly higher in patients with moderate (*p* < 0.01) and high (*p* < 0.001) DAS28 scores in comparison to the healthy controls. Moreover, TLR4 expression significantly increased (*p* < 0.001) between the three groups of patients with low DAS28 scores to those with high scores showing a potential clinical relevance of TLR4 cell surface expression on CD8^+^ T cells of RA patients.

A Spearman non-parametric correlation analysis was performed to further confirm the identified clinical relevance of TLR4 protein (Fig. 2c). Interestingly, the TLR4 expression correlated linearly with the DAS28 scores (*r*_*s*_ = 0.96, *p* < 0.0001) representing a significant progression of TLR4 expression in CD8^+^ T cells with disease progression towards severity.

The ROC curve generated to determine the sensitivity and the specificity of the TLR4 expression on CD8^+^ T cells of RA patients in comparison to the healthy controls further demonstrates that TLR4 levels on CD8^+^ T cells are accurately distinguishing patients with RA from the healthy individuals (Area = 0.77, *p* < 0.01) (Fig. 2d).

### Distinct subtypes of CD8^+^ T cells in RA express TLR4 and have a potent cytolytic and inflammatory profile

**Figure 3:**
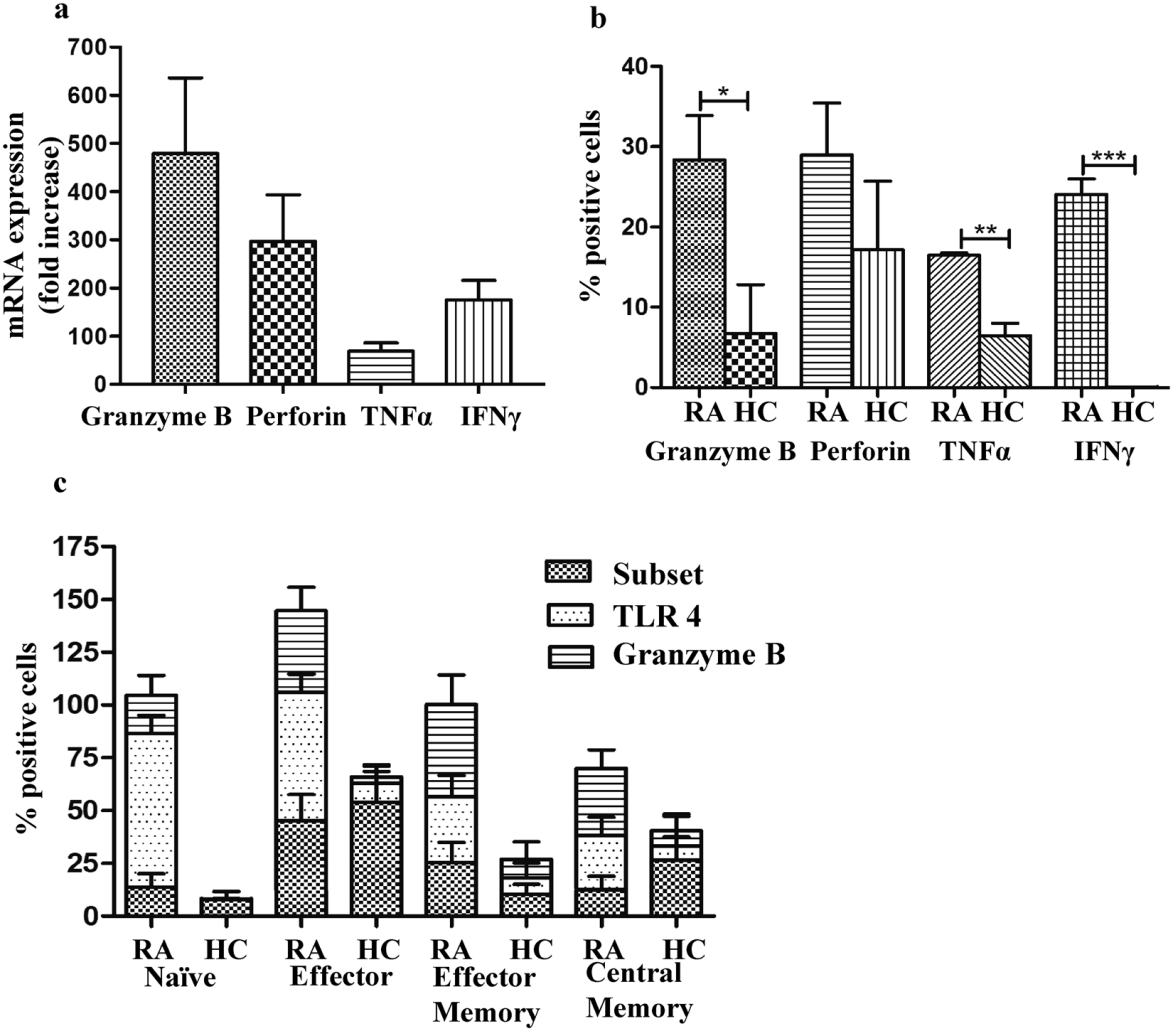
Inflammatory mediators profiling of CD8^+^ T cell in RA patients. (**a**) The figure represents fold increase in the relative mRNA expression, calculated with respect to β-actin, for Granzyme B, Perforin, TNFα and IFNγ in CD8^+^ T cells isolated from RA patients (n = 23) and HC samples (n = 23). (**b**) Bar graph shows percentage of Granzyme B, Perforin, TNFα and IFNγ expressing CD3^+^CD8^+^ T cells in rheumatoid arthritis (RA, n = 5) and healthy control (HC, n = 4) samples. (**c**) Bar graph represents CD8^+^ T cell subpopulations and a co-expression of TLR4 and Granzyme B proteins (n = 5, RA and n = 4, HC). Bars represent the mean ± SEM. *p < 0.05, **p < 0.01, ***p < 0.001.

We next sought to determine differences in the cytolytic and inflammatory potential of CD8^+^ T cells of RA patients from those of healthy individuals. For this, Granzyme B, Perforin, TNFα and IFNγ transcript expression was analyzed in *ex vivo* isolated peripheral CD8^+^ T cells using qPCR and protein expression was analyzed with flow cytometry. Interestingly, we observed significant expression of these molecules both at the mRNA transcript and protein levels. A robust increase in mRNA transcripts and increased average fold changes of mRNA transcripts of Granzyme B (479.4 ± 159.7 fold), Perforin (296.5 ± 96.9 fold), TNFα (68.3 ± 17.7 fold), and IFNγ (175.4 ± 40.26 fold) was observed in RA patients as compared to healthy controls (Fig. 3a). A concomitant increase in the expression of these proteins on CD8^+^ T cells was observed in RA patients. A higher percentages of CD8^+^ T cells in RA patients expressed Granzyme B (RA: 28.40 ± 5.5% vs HC: 6.73 ± 6.0%; p < 0.05), Perforin (RA: 28.97 ± 6.5% vs HC: 17.17 ± 8.5%), TNFα (RA: 16.47 ± 0.3% vs HC: 6.47 ± 1.5%; p < 0.01) and IFNγ (RA: 24.07 ± 1.9% vs HC: 0.03 ± 0.01%; p < 0.001) was observed in RA patients in comparison to the healthy controls (Fig. 3b and Supplementary Fig. 2). The results obtained are in accordance to the previous report wherein CD8^+^ T cells of active RA patients were shown to produce mediators of cytotoxicity including Granzyme B, Perforin, TNFα and IFNγ^28^. The study also reported that RA patients have a selective increase in the CD27^−^CD62L^−^CD8^+^ effector subpopulation of CD8^+^ T cells. In order to extrapolate these findings in our case-control cohort we examined the spread of CD8^+^ T cell subpopulations in RA patients and healthy controls using flow cytometry (Supplementary Fig. 1c). However, we did not notice any significant difference in the percentages of CD8^+^CD45RO^−^CD28^−^ effectors, CD8^+^CD45RO^+^CD28^−^ effector memory, CD8^+^CD45RO^+^CD28^+^ central memory or CD8^+^CD45RO^−^CD28^+^ naïve T cells (Fig. 3c) among RA patients and the healthy donors^18^. Thus we may conclude that in the present cohort the CD8^+^ T cell subpopulation is not variable.

It is interesting to observe that though CD8^+^CD45RO^−^CD28^−^ effector lymphocytes have not selectively proliferated in the RA patients yet CD8^+^ T cells were highly activated. Thus in order to confirm the role of TLR4 in the effector-like function of CD8^+^ T cells we used flow cytometry to analyze the co-expression of TLR4 and Granzyme B on different subpopulations of CD8^+^ T cells of RA patients and healthy controls. In contrast to the healthy controls that did not express much TLR4 receptor on their CD8^+^ T cells, RA patients expressed increased TLR4 in effector (8.92 ± 8.9% in HC vs 60.76 ± 8.7% in RA), naïve (0.28 ± 0.2% in HC vs 72.77 ± 8.4% in RA), effector memory (7.52 ± 7.0% in HC vs 30.92 ± 10.2% in RA) and central memory (6.74 ± 4.3% in HC vs 25.75 ± 8.7% in RA) subpopulations of CD8^+^ T lymphocytes (Fig. 3c). Moreover, all these CD8^+^ T cell subsets co-expressed Granzyme B. The significant differences in Granzyme B expression were observed in effector (38.73 ± 11.2% cells) lymphocytes of RA patients (Fig. 3c) while expression level for Granzyme B in the healthy controls remained very low (3.125 ± 2.63% cells). Thus the above data may indicate, however requires further investigations, a probable presence of TLR4 on CD8^+^ T cells of RA patients that may be involved in the activation of CD8^+^ T lymphocytes and eventually promoting CD8^+^ T cell mediated cytotoxicity and inflammatory environment in RA patients.

### TLR4-dependent activation of CD8^+^ T cells from RA patients augments cytolytic and inflammatory lymphocyte responses

**Figure 4:**
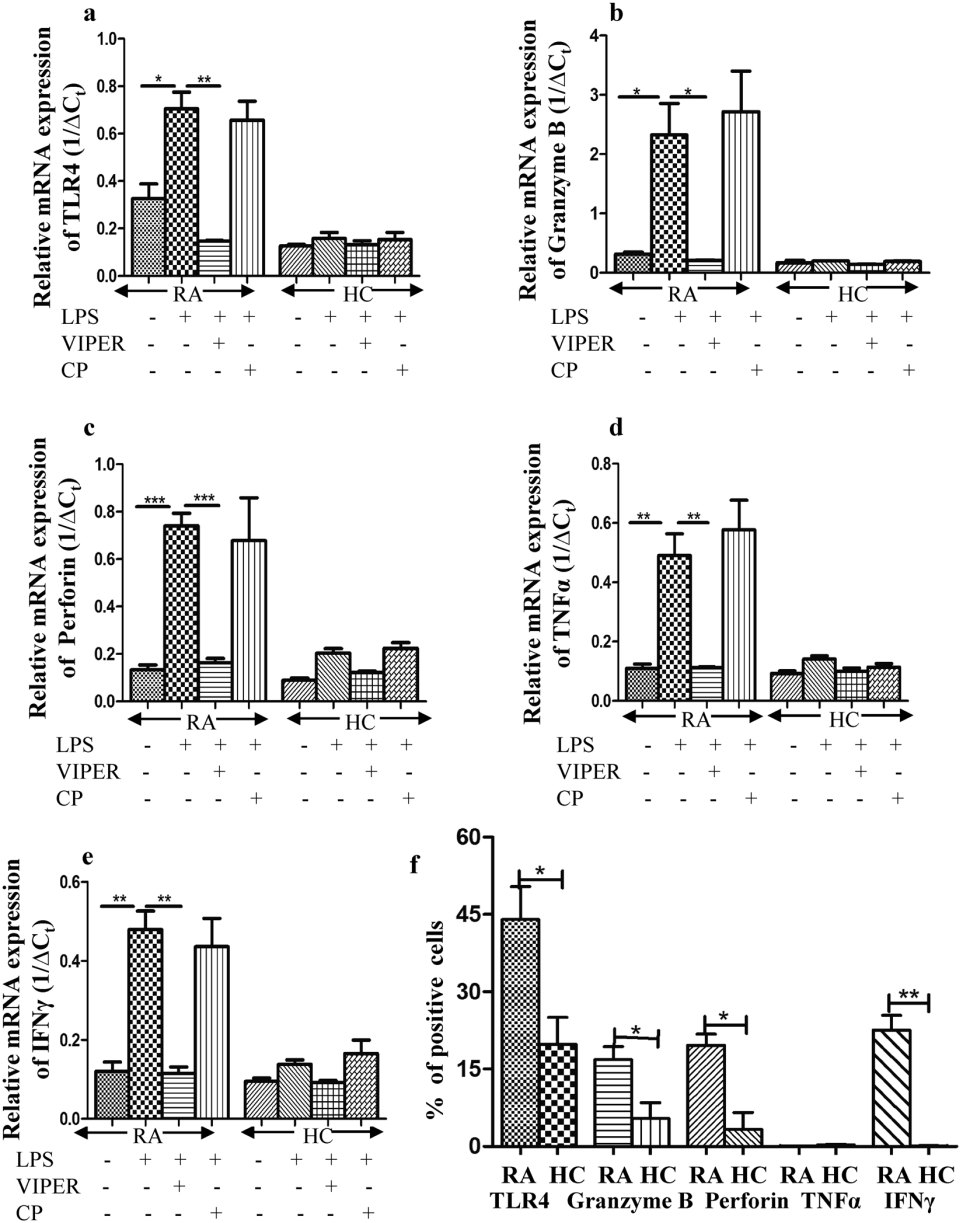
LPS triggers TLR4 dependent activation of CD8^+^ T cells isolated from RA patients augmenting cytotoxic and inflammatory lymphocyte responses. CD8^+^ T cells isolated from RA (n = 6) and HC (n = 6) blood samples were pre-stimulated with 5 µM of VIPER and 5 µM CP7 for 2 hours and then treated with 5 µg/ml LPS for 6 hours. These cells were then evaluated for relative mRNA expression with respect to β-actin for TLR4 (**a**), Granzyme B (**b**), Perforin (**c**), TNFα (**d**) and IFNγ (**e**). Pre-stimulation of VIPER significantly inhibited the expression of all mediators while the inert peptide CP7 had no effect. (**f**) The graph shows percent CD8^+^ T cells expressing TLR4, Granzyme B, Perforin, TNFα and IFNγ proteins in the experimental set up. Bars represent the mean ± SEM. *p < 0.05, **p < 0.01, ***p < 0.001.

We found that TLR4 is expressed on CD8^+^ T cells of RA patients but the functional significance of this is unclear. In line with our findings; we next examined if CD8^+^ T cells of RA patients respond to a TLR4 ligand and generate a cytotoxic and inflammatory response. To this end, we performed a time course analysis using CD8^+^ T cells isolated from RA patients (n = 6) and healthy controls (n = 6) stimulated with different concentrations of LPS, a potent TLR4 ligand (Supplementary Fig. 3). Within 6 hours, these T lymphocytes of RA patients showed robust mRNA expression of TLR4, Granzyme B, Perforin, TNFα and IFNγ transcripts when stimulated with 5 µg/ml LPS, with only marginal expression in lymphocytes from healthy controls (Fig. 4a–e and Supplementary Fig. 3).

In order to confirm that the observed effect is specifically TLR4 mediated we performed inhibition experiments using a TLR4 inhibitor, the VIPER peptide. Using 5 µM VIPER peptide, TLR4 mediated Granzyme B, Perforin, TNFα, and IFNγ production was abrogated in all the experiments (Fig. 4a–e). CP7 was always included as a negative control peptide for all inhibition assays.

We further confirmed these observations by analyzing the expression of these proteins by flow cytometry. A significant increase (p ≤ 0.05) in the number of CD8^+^ T cells expressing surface TLR4 (RA: 44.00 ± 6.4% vs HC: 19.78 ± 5.3%), Granzyme B (RA: 16.83 ± 2.6% vs HC: 5.4 ± 3.06%), Perforin (RA: 19.60 ± 2.2% vs HC: 3.31 ± 3.27%) and IFNγ (22.53 ± 2.9% vs HC: 0.14 ± 0.04%) was observed, post stimulation with LPS, in RA patients in comparison to those from healthy individuals (Fig. 4f and Supplementary Fig. 4). We could not detect expression of TNFα in these experiments.

Taken together our study supports an important role of this innate receptor in the direct regulation of CD8^+^ T cell activation in RA.

## Discussion

CD8^+^ T cells are shown to be abundant in RA^40^ however their role in the pathogenesis and progression of RA is poorly defined. The presence of different subpopulations of CD8^+^ T cells, their increased cytotoxic and pro-inflammatory behavior in RA has been reported previously^28^ and in line with this report^28^, we as well identified cytolytic and activated CD8^+^ T cells in RA patients. These cells expressed significant levels of mRNA transcripts for cytolytic molecules like Granzyme B and Perforin as well as inflammatory mediators like TNFα and IFNγ (Fig. 3). A significant increase in the number of cells expressing these proteins was also observed in RA patients. This suggests that the CD8^+^ T lymphocytes in RA have an activated effector-like function and play a significant role in promoting inflammatory responses. However, we could not find a selective expansion of any subpopulation of these CD8^+^ T cells in RA patients. The number of effectors and memory cells were equally distributed in both the patient and control individuals. The discrepancy of similar spread of CD8^+^ T cell subpopulation in RA patients and the healthy controls can be explained well as the T cell proliferation and cytokine expression is reported to be not always correlated^34^. However although not significant we did notice a small trend of increased number of effector memory CD8^+^ T cell in RA patients (Fig. 3). It was thus necessary to understand the specific mechanisms leading to increased cytotoxicity and inflammatory responses of these T lymphocytes in RA.

Unconventional presence of TLRs on the surface of CD8^+^ T cells has opened new avenues to understand the mechanisms of CD8^+^ T cell activation and function in autoimmune diseases like RA. TLRs have been largely involved in triggering innate immune responses and priming antigen-specific adaptive immunity. TLR4 is a specific exogenous receptor for LPS, which plays a vital role in pathogen recognition and activation of the innate immune system. TLR4 is one of the most extensively studied TLRs involved in autoimmune diseases. A number of studies have suggested that TLR4-mediated inflammation may play a critical role in the development of RA^41^. Roelofs *et al*. reported that TLR4 can mediate synovial inflammation in RA by its expression on both macrophages and fibroblasts of the synovial tissue^42^. Interestingly, we found TLR4 expression on CD8^+^ T cells present in the synovial fluid of RA patients. TLR4 signaling was also proposed to enhance and sustain inflammation thereby contributing to pathogenesis of RA^43^. Recently it was also reported that human CD8^+^ T cells express functional LPS receptor complex and can directly recognize LPS^44^. In light of these reports we hypothesized a TLR4 mediated activation of CD8^+^ T cells in RA patients.

To begin with, we observed significantly increased mRNA expression of TLR4 on CD8^+^ T cells in RA patients. We also found an increased number of CD8^+^ T cells expressing TLR4 on their surface as well as an extensive expression of TLR4 receptors on each CD8^+^ T cells of RA patients (Fig. 1). Moreover all subpopulations of T cells expressed varying but significant levels of TLR4 on their membranes and secreted robust amounts of Granzyme B (Fig. 3). We thus confirmed that TLR4 expression on CD8^+^ T cells of RA patients has a potential role in T cell activation and or function.

Pathogenesis of RA has been long debated to be an effect of different microbial species. Differences in the gut micro biome of RA patients leading to pathogenesis and progression of RA have been recently suggested^45^. Moreover, RA patients have a reservoir of auto-antigens that can act to engage TLRs and activate TLR dependent inflammation^38, 39^. Growing evidence suggests that T cells participate in immune microbial surveillance^34, 44^ and our finding that the TLR4 expression correlated significantly with the severity of the disease (DAS28 scores) and that CD8^+^TLR4^+^ T cells also secrete cytolytic molecules suggested a possible direct effect of TLR4 ligand on T cells. We thus challenged CD8^+^ T cells isolated from RA patients with LPS, a known ligand for TLR4. Interestingly, TLR engagement with LPS spontaneously stimulated CD8^+^ T cells from RA patients and activated them for a significant production of cytolytic molecules like Granzyme B and Perforin as well as the inflammatory mediators including TNFα and IFNγ (Fig. 4). These data further demonstrate that TLR4 signals directly drive CD8^+^ T cell activation and Tc1 development in RA patients and suggest that some components from invading or resident microorganisms as well as auto-antigens can directly stimulate T cells in RA patients by up-regulating productions of cytolytic molecules and cytokines. Moreover, an infection can result in molecular mimicry by cross-reactivity of pathogen-specific CD8^+^ T cells to self-antigens with structural similarity. CD8^+^ T cells in these patients expressing elevated IFNγ may thus contribute to disease progression.

We report here for the first time that RA patients express functional TLR4 on peripheral CD8^+^ T cells that directly promote T-cell function and differentiation to Tc1. The present study also suggests that TLR4 signals directly drive Tc1 development and T cell activation independent of TCR engagement. While the mechanism is still unclear and currently under investigation, this is in agreement with the previous findings that TLR agonists selectively stimulate IFNγ but not IL-4 production by human γδ T cells^19, 22, 28^. The TCR independent T cell activation directly by TLR4 explains that TLR ligands besides influencing the adaptive immune response through APC activation can also drive polyclonal activation and differentiation of antigen-specific CD8^+^ T cells which may impact on the infection-exacerbated inflammatory and autoimmune diseases like RA. However, this may also represent a relevant pathway for the host to mount an immediate robust type I response during an infection or re-infection. Moreover, it has been previously reported that activated CD8^+^ T cells contribute to DC activation thereby inducing Th1-promoting phenotype in DCs^46^ thus CD8^+^ T cells in RA may have a competent role by acting as “helper cells” for the induction of Th1-polarized inflammatory-type responses. Also these CD8^+^ T cells may further sustain this Th1 response during their interaction with DCs by supporting the differentiation of monocytes into Th1-inducing Tip-DCs^47^ in RA. Thus the present work opens up new perspectives and highlights CD8^+^ T cells as a critical modulator of disease that has been largely overlooked.

## Materials and Methods

### Tissue collection

One hundred and three RA patients participated in this study were followed at the Rheumatology outpatient clinic of the Pradyumna Bal Memorial Hospital, Bhubaneswar, India. Blood samples were obtained from the healthy donors and the patients who met the 2010 ACR/EULAR classification criteria for the diagnosis of RA^48^. Clinical variations like disease duration, number of actively inflamed joints, number and type of bones deformities, CRP, RF, Anti-CCP, ESR (Erythrocyte Sedimentation Rate) and therapeutic interventions were recorded for each patient and DAS 28 scores were calculated with the help of the clinician (Table 1). Informed consent was obtained from all donors and the study was approved by the Institute and Hospital Research Ethics Committee and all methods were performed in accordance with the institutional guidelines and regulations.

**Table 1:** Characteristics of 103 patients with rheumatoid arthritis included in the study.

Clinical and para-clinical Variables	‘Low’ RA (n = 35) Mean ± SD	‘Moderate’ RA (n = 42) Mean ± SD	‘High’ RA (n = 26) Mean ± SD
Gender (F/M)	25/10	32/10	14/12
Age (years)	42 ± 6.5	40 ± 7.0	50 ± 8.5
Disease duration (years)	2 ± 1.6	3 ± 1.3	5 ± 2.4
DAS	≤4	6.3 ± 0.14	9.0 ± 0.1
Swollen joint count	5.3 ± 3.6	6.4 ± 1.2	10 ± 4.2
Tender joint count	6.5 ± 3.8	10.2 ± 2.1	14.3 ± 1.2
ESR (mm/1^st^ h)	30.1 ± 2.7	60.2 ± 3.6	82.9 ± 3.4
CRP (mg/L)	12.5 ± 0.7	36.5 ± 1.3	50.6 ± 4.7
RF positive (%)	100	100	100
Anti-CCP (%)	100	100	100

DAS: disease activity score, ESR: erythrocyte sedimentation rate, CRP: C-reactive protein, RF: rheumatoid factor, CCP: cyclic citrullinated peptide.

### CD8^+^ T cells isolation and purification

Human peripheral blood samples were collected in lithium heparin coated vacutainers (BD Vacutainer, 367886) from patients and healthy donors. Three synovial fluid samples were also collected under sterile conditions by the clinicians. CD8^+^ T cells were then isolated from blood and synovial fluid samples using RosetteSep Human CD8^+^ T Cells enrichment cocktail (StemCell Technologies Inc, BC, Canada) followed by Ficoll-Hypaque density gradient (HiSepTM LSM 1077, HIMEDIA, India) according to manufacturer’s protocol. Isolated plasma was stored in −80 °C for future use. Purity of isolated CD8^+^ T cells from blood samples was evaluated by flow cytometry (BD LSRFortessa) using FITC-anti-CD3 and PE-anti-CD8a antibodies (TONBO biosciences, San Diego, CA) and FACS Diva or FlowJo V10 software (Supplementary Fig. 1a). For flow cytometric analysis minimum of 10,000 viable events (CD8^+^ T cells) were acquired per sample.

### Quantitative PCR analysis

Total RNA was extracted by TRIzol reagent (Life Technologies Inc, USA) according to manufacturer’s protocol and quantified by Nano drop (Thermo Scientific). Single strand cDNA was synthesized by High-Capacity c-DNA Reverse Transcription Kit (AB Applied Biosystem, CA, USA). Quantitative real-time PCR reactions were prepared with SYBR green master mix I(Roche, IN, USA) in 384 well plates using Liquid handling system (epMotion 5075, Eppendorf) and real-time PCR was performed in triplicate with Light Cycler 480 II (Roche). The sequences of primers used are listed in Table 2. The Ct values obtained from each gene were normalized with the housekeeping gene ß-actin expressed as ∆C_t_.

**Table 2:** List of primers used for quantitative real time PCR.

Primer	Forward Sequence	Reverse Sequence
TLR4	5′AAGAAGGGGTGCCTCCAT3′	5′CCACACCGGGAATAAAGTCTC3′
Granzyme B^49^	5′GCAGGAAGATCGAAAGTGCGA3′	5′GCATGCCATTGTTTCGTCCAT3′
Perforin^50^	5′CGCCTACCTCAGGCTTATCTC3′	5′CCTCGACAGTCAGGCAGTC3′
TNFα^51^	5′GGAGAAGGGTGACCGACTCA3′	5′CTGCCCAGACTCGGCAA3′
IFNγ^51^	5′CCAACGCAAAGCAATACATGA3′	5′CCTTTTTCGCTTCCCTGTTTTA3′
ß-actin	5′GCTACGAGCTGCCTGAGC3′	5′GGCTGGAAGAGTGCCTCA3′

### Flow cytometry

CD8^+^ T cells isolated as above were washed with FACS buffer (PBS containing 2% FBS and 2 mM EDTA) and labeled for 30 min in dark at 4 °C with the following monoclonal antibodies (TONBO biosciences) anti-CD3-FITC, anti-CD8a-PE, anti-TLR4-PE Cy7 (eBioscience, CA, USA), anti-CD28-PerCpCy5.5, anti-CD45RO-FITC(TONBO biosciences). After surface staining with anti-TLR4-PECy7 antibody, CD8^+^ T cells were then fixed for 30 min with 2% PFA, permeabilized with Perm buffer (TONBO biosciences) and labeled with anti-Granzyme B-PE (eBioscience), anti-Perforin-BV 421, anti-TNFα-PE, anti-IFNγ-APC/Alexa Fluor 700 antibodies (BD Biosciences). Expression levels were measured using BD LSRFortessa (BD Biosciences), and data were analyzed using FlowJo software (version 10; Tree Star). CD3^+^CD8^+^ T cells, directly gated in whole blood, (Supplementary Fig. 1b) were also analyzed for TLR4 surface protein expression.

### *Ex vivo* CD8^+^ T cell activation

Viability of purified CD8^+^ T cells was analyzed using Trypan blue (Life technologies, CA, USA) and automated cell counter (Countess, Life technologies). A total of 50,000 viable cells/well were cultured in 200 ul of lymphocyte culture media (10% FBS, 0.5 mM 2-Mercaptoethanol, 1% L-Glutamine-penicillin-streptomycin solution in RPMI 1640 (Thermo Fisher Scientific, MA, USA) in 96-well flat-bottom cell culture plates (NEST). Cells were stimulated with media alone, 1 µg/ml or 5 µg/ml of LPS-EB ultrapure (InvivoGen, CA, USA) separately for 2 hours and 6 hours at 37 °C and 5% CO_2_. The mRNA from the cells was extracted using TRIzol reagent and qPCR was performed as described above. For TLR4 inhibition experiments, cells were pre-stimulated with 5 µM or 3 µM concentration of TLR4 inhibitor peptide VIPER (NovusBio, CO, USA) and the equal concentration (5 µM or 3 µM) of control peptide CP7 (NovusBio) for 2 hours at 37 °C and 5% CO_2_. The cells were then treated with 5 µg/ml LPS for 6 hours. For all subsequent experiments we continued using 5 µM of inhibitory and control peptide for 2 hours followed by incubation with 5 µg/ml of LPS-EB ultrapure for 6 hours. Expression of different mRNA transcript of inflammatory mediators was analyzed with qPCR and protein expression of Granzyme B, Perforin, TNFα, IFNγ and TLR4 was analyzed using flow cytometry. For flow cytometry experiments we have added 10 ug/ml of secretion inhibitor Brefeldin A (eBioscience) along with LPS.

### Statistics

Statistical analysis was performed using Graph-Pad Prism 5. The unpaired t-test was used to compare mRNA expression as well as protein expression between Rheumatoid arthritis patients and healthy controls. Spearman non parametric analysis was used to analyze the correlation between two factors. ROC and area under the curve was determined by Graph pad prism 5. A two-tailed *p* value of <0.05 was considered to indicate significance.

## Electronic supplementary material

Supplementary Info
