# Supplementary material for: Direct recognition of LPS drive TLR4 expressing CD8+ T cell activation in patients with rheumatoid arthritis
Source: Sci Rep. 2017 Apr 19;7:933. doi: 10.1038/s41598-017-01033-7 (PMC5430440; doi:10.1038/s41598-017-01033-7)
Supplement: Supplementary file 1 — Supplementary Info [file 41598_2017_1033_MOESM1_ESM.pdf]

**Direct recognition of LPS drive TLR4 expressing CD8$^{+}$ T cell activation in patients with rheumatoid arthritis.**

Archana Tripathy[1]*, Shweta Khanna[1]*, Prasanta Padhan[2], Shuchi Smita[3], Sunil Raghav[3] and Bhawna Gupta[1#].

**Supplementary Figures:**

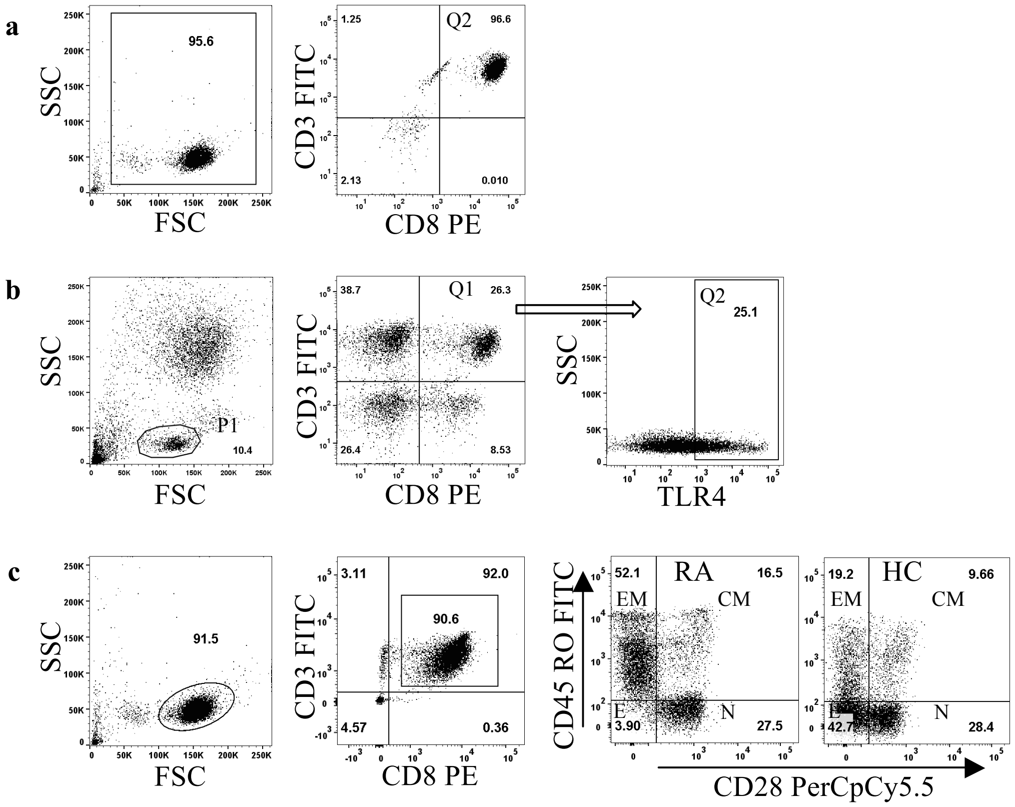


**Supplementary Figure 1:** (a) Flow cytometry gating strategy for isolated CD8$^{+}$ T cells using RosetteSep CD8$^{+}$ T cells isolation kit. Quadrant 2 (Q2) represents percentage of CD3 and CD8 double positive cells from enriched samples. (b) Flow cytometry gating strategy for identification of TLR4 expressing CD3$^{+}$CD8$^{+}$ T cells from PBMCs. Lymphocyte population was gated and named as P1. Q1 population represents CD3$^{+}$ CD8$^{+}$ T cells and Q2 population shows TLR4$^{+}$ CD3$^{+}$CD8$^{+}$ T cells. (c) Gating strategy for identification of CD8$^{+}$ T cells subpopulations (N: Naïve cells, E: Effector cells, EM: Effector memory cells, CM: Central memory cells).

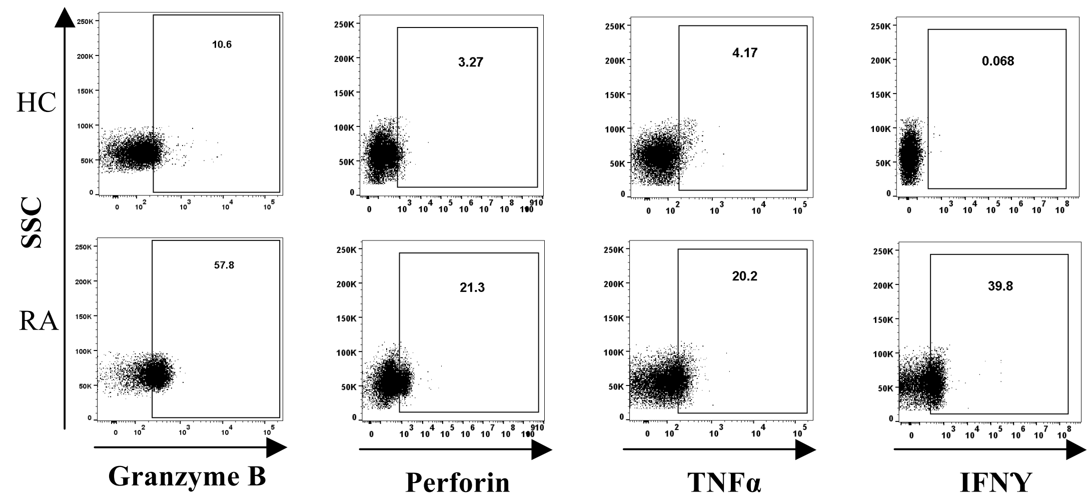


**Supplementary Figure 2:** Dot plots show a representative figure of Granzyme B, Perforin, TNFα and IFNγ expressing $CD8^+$ T cells in a HC and RA patient. Upper panels represent for healthy control and lower panels represent for RA patients.

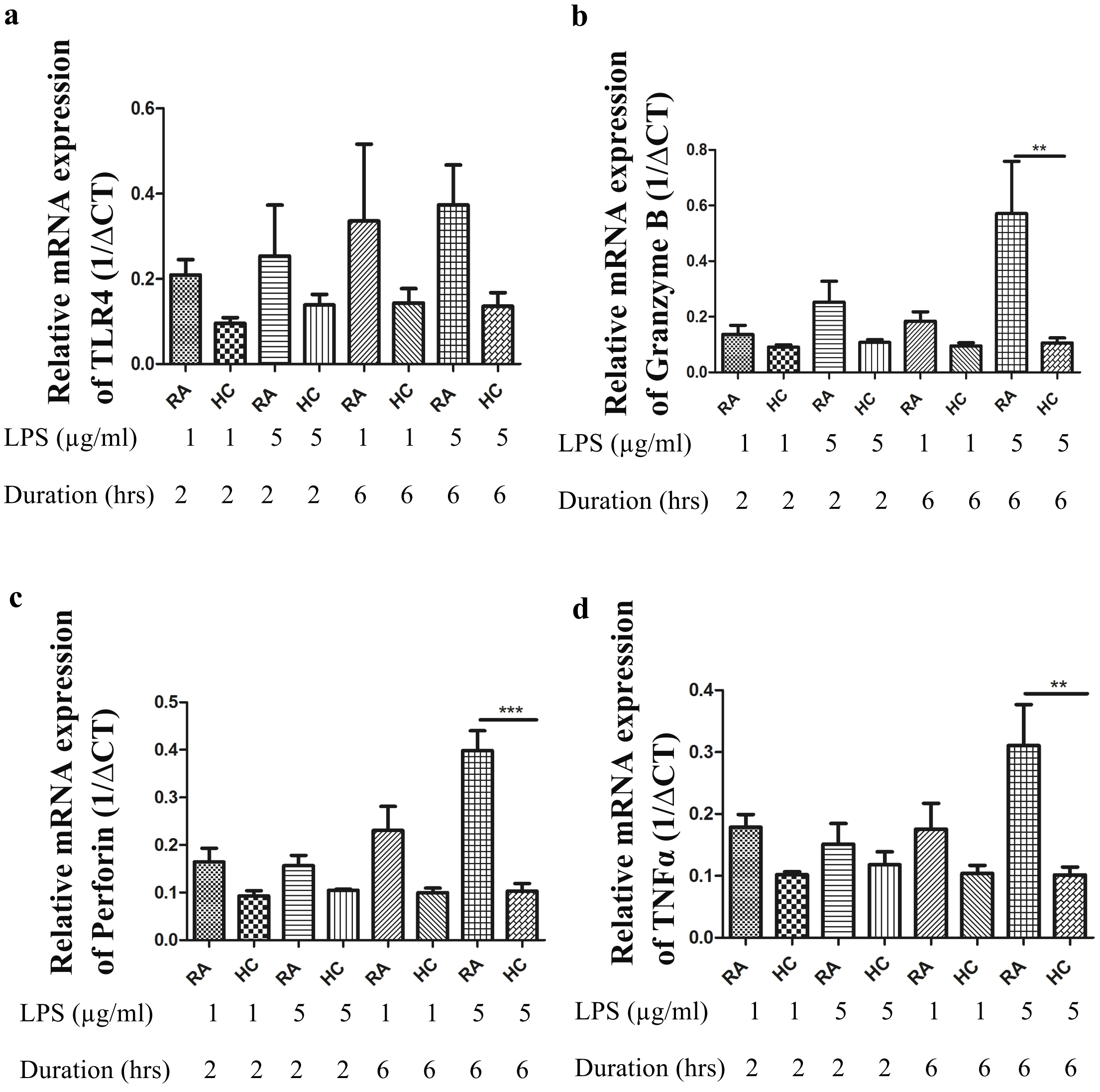

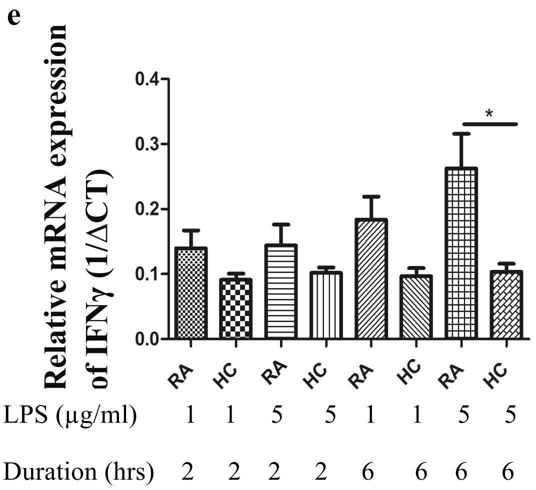


**Supplementary Figure 3:** Different stimulation conditions of CD8+ T cells isolated from RA patients to observe expression pattern of TLR4, Granzyme B, Perforin, TNFα and IFNγ mRNA transcripts. CD8+ T cells were isolated from both RA patients (n=6) and healthy controls (n=6) and cultured for 2hrs and 6hrs with 1 μg/ml and 5 μg/ml LPS in RPMI-1640 media. Results are expressed as normalized RNA values ($1/\Delta C_t$) at 2hrs and 6hrs after culture. (b, c, d, e). A significant increase in mRNA expression of Granzyme B, Perforin, TNFα and IFNγ in CD8+ T cells of RA patients incubated with 5μg/ml concentration of LPS for 6hrs in comparison to HC was observed. Bars represent mean ± SEM. * $p<0.05$, ** $p<0.01$, *** $p<0.001$.

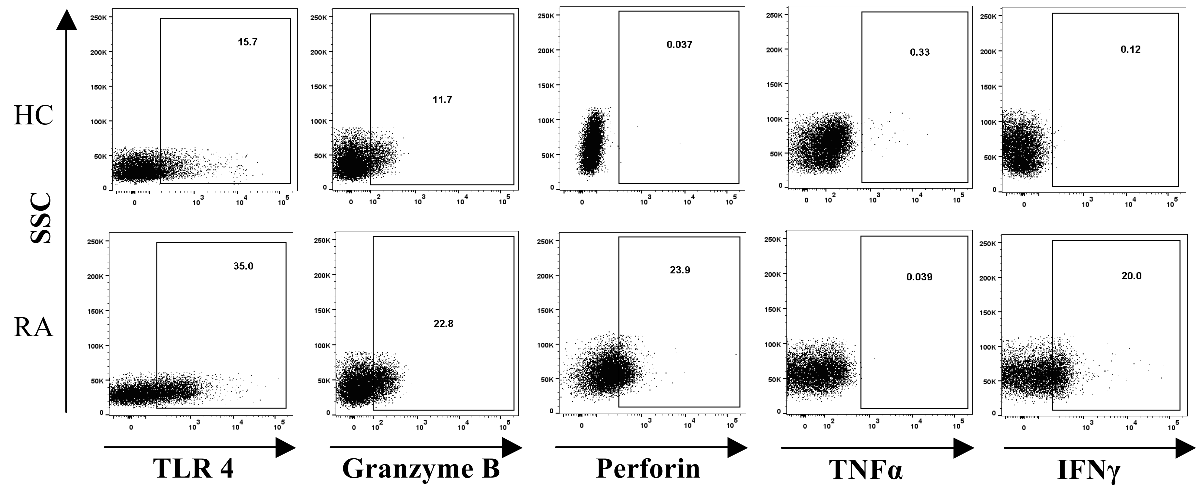

**Supplementary Figure 4:** Representative dot plots for TLR 4, Granzyme B, Perforin, TNFα and IFNγ expressing $CD8^+$ T cells after stimulating with 5 μg/ml of LPS-EB ultrapure for 6 hrs at 37°C and 5% $CO_2$ in a HC and RA patient. Upper panels represent healthy control and lower panels represent RA patient.
